# Genomic Analysis of Colombian *Leishmania panamensis* strains with different level of virulence

**DOI:** 10.1038/s41598-018-35778-6

**Published:** 2018-11-26

**Authors:** Daniel Alfonso Urrea, Jorge Duitama, Hideo Imamura, Juan F. Álzate, Juanita Gil, Natalia Muñoz, Janny Alexander Villa, Jean-Claude Dujardin, José R. Ramirez-Pineda, Omar Triana-Chavez

**Affiliations:** 1 Grupo Biología y Control de Enfermedades Infecciosas, Universidad de Antioquia, Medellín, Colombia; 2 Systems and Computing Engineering Department, Universidad de los Andes, Bogotá, Colombia; 3 Institute of Tropical Medicine, Antwerpen, Belgium; 4 Centro Nacional de Secuenciación Genómica, Universidad de Antioquia, Medellín, Colombia; 5 Grupo Inmunomodulación, Universidad de Antioquia, Medellín, Colombia; 6 Laboratorio de Investigaciones en Parasitología Tropical (LIPT), Departamento de Biología, Facultad de Ciencias, Universidad del Tolima, Tolima, Colombia

## Abstract

The establishment of Leishmania infection in mammalian hosts and the subsequent manifestation of clinical symptoms require internalization into macrophages, immune evasion and parasite survival and replication. Although many of the genes involved in these processes have been described, the genetic and genomic variability associated to differences in virulence is largely unknown. Here we present the genomic variation of four *Leishmania (Viannia) panamensis* strains exhibiting different levels of virulence in BALB/c mice and its application to predict novel genes related to virulence. *De novo* DNA sequencing and assembly of the most virulent strain allowed comparative genomics analysis with sequenced *L. (Viannia) panamensis* and *L. (Viannia) braziliensis* strains, and showed important variations at intra and interspecific levels. Moreover, the mutation detection and a CNV search revealed both base and structural genomic variation within the species. Interestingly, we found differences in the copy number and protein diversity of some genes previously related to virulence. Several machine-learning approaches were applied to combine previous knowledge with features derived from genomic variation and predict a curated set of 66 novel genes related to virulence. These genes can be prioritized for validation experiments and could potentially become promising drug and immune targets for the development of novel prophylactic and therapeutic interventions.

## Introduction

Leishmaniasis is a group of neglected tropical diseases caused by parasites belonging to the *Leishmania* genus, affecting around 14 million people worldwide and with 350 million people at risk of infection. There are three main forms of the disease: cutaneous leishmaniasis (CL), mucocutaneous leishmaniasis and visceral leishmaniasis^1–3^. Whether one of those clinical forms or the asymptomatic infection is developed, depends on a complex interaction between host- and parasite-derived factors. A better understanding the mechanisms of pathogenesis and immunity in Leishmania infections is crucial for the study of parasite-host interaction and the development of new therapies and vaccines for leishmaniasis.

Comparative genomic analysis is a powerful tool to discover genetic features that might underlie the variability in pathogenesis and clinical manifestations among different forms of leishmaniasis. Genome comparison between *Leishmania donovani* and *L. major* allowed to identify genes involved in virulence and tissue tropism after infections in animal models^4,5^. Genome sequencing has also allowed the identification of genes associated with the intracellular amastigote stage in the pathogenic Leishmania species^6^. Similarly, whole genome sequencing (WGS) confirmed the deletion of virulence genes in genetically modified strains of *L. donovani* with attenuated virulence^7^. Moreover, genomic variations such as aneuploidies, single nucleotide polymorphisms (SNPs) and structural variants (SVs) such as copy number variation (CNV) can affect the presence, dosage, and consequently the expression of alleles of genes related to virulence. SNPs, CNVs and aneuploidies were suggested as drivers of tropism towards cutaneous or visceral tissue and virulence in *L. donovani*^8^. In addition, WGS also showed differences in SNPs between strains of *Trypanosoma brucei* that generate chronic or acute infections^9^.

Recent years, the importance of parasite virulence factors has become evident. Supplementary Table S1 shows a list of 94 genes reported to be involved in virulence or up-regulated in the amastigote stage. Although these genes have been experimentally associated to processes necessary to establish infection and response to different pressures or stress, the variability of these genes among and within Leishmania species is largely unknown.

Leishmania species belonging to the Viannia subgenus, such as *Leishmania (Viannia) panamensis*, are major causal agents of American cutaneous leishmaniasis (ACL). A wide spectrum of clinical manifestations caused by this group of parasites has been reported in humans^1,10^ and animal models^11^. This variation is attributed to variability not only in the host immune response but also in parasite virulence^12–16^. To investigate the mechanisms that mediate virulence in the ACL caused by *L. panamensis*, we report here the genome of the virulent UA946 strain, and the genomic variability between this and another three *L. panamensis* strains exhibiting different levels of virulence in BALB/c mice. Moreover, we compare the assembled genome with the previously reported draft genome for the species and the Viannia reference genome *L. braziliensis*. Following machine learning approaches we predict new possible genes involved in virulence. Our results suggest that differences in dosage of some genes involved in virulence and allelic diversity through single nucleotide mutations may be determinant in the level of virulence of these strains in the murine model.

## Results

### *L. panamensis* strains exhibit different levels of virulence in BALB/c mice

**Figure 1:**
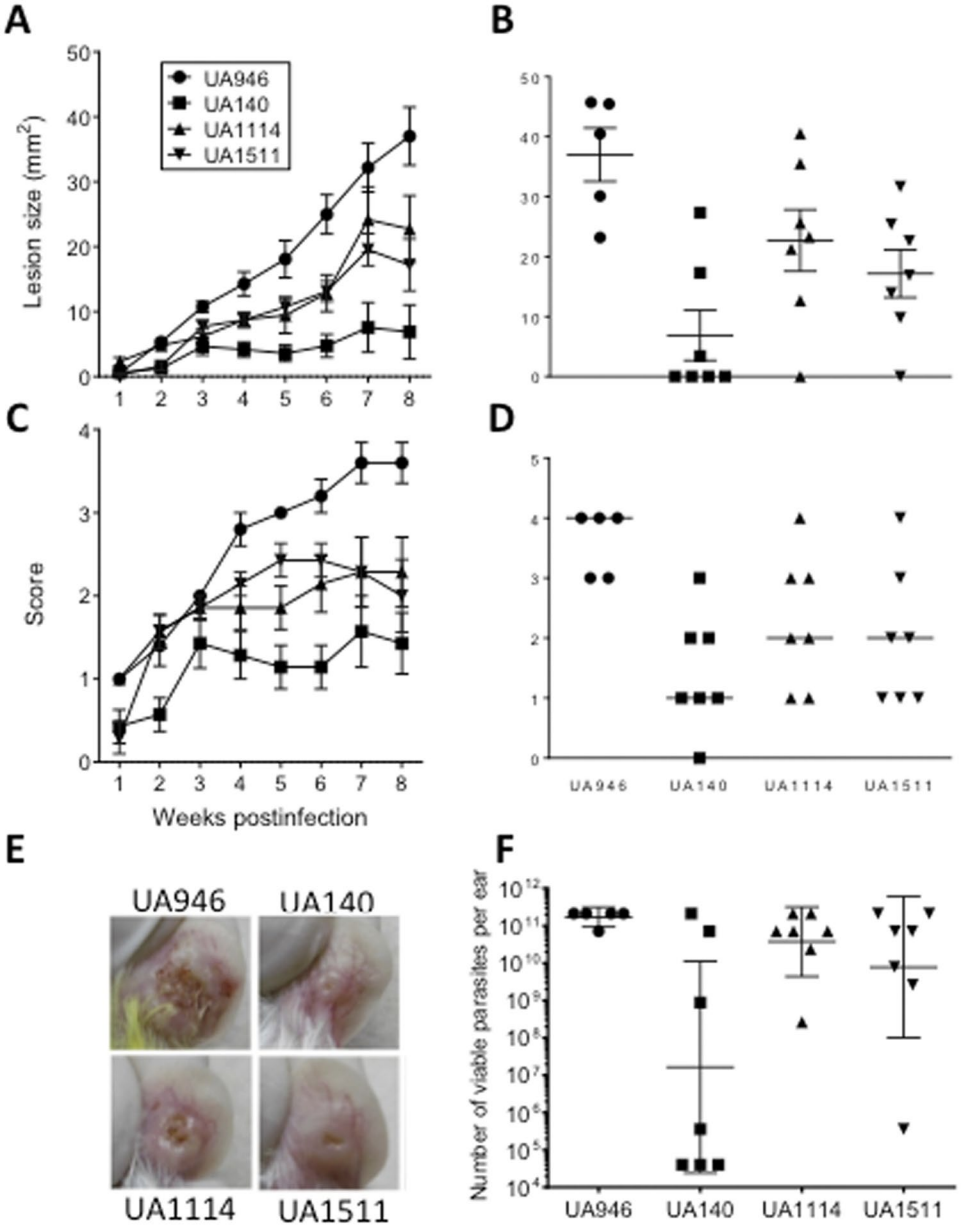
Virulence of four *L. panamensis* strains in BALB/c mice. BALB/c mice were infected as described in material and methods and the size of the lesion was determined weekly (**A**) or at the 8^th^ week post-infection and reported in individually (**B**). The severity score was also monitored weekly (**C**) or 8 weeks post-infection in individual mice (**D**). Representative photographs of the infected ears at the end of the experiment (8^th^ week post-infection) are presented for each experimental group (**E**). Parasitic loads records (**F**). Graphs show the mean+/−SEM (**A**,**B**,**C**), the median (**D**) or the geometric mean +/− 95% CI (**F**).

The availability of a collection of *L. panamensis* strains with a range of virulence levels in mice, motivated us to perform the genomic comparative study reported here. We began by comparing four strains together in the same experiment. All BALB/c mice infected with UA946 develop lesions that progressed to large ulcers within 8 weeks, whiles mice infected with UA140 develop no lesion or a mild disease (Fig. 1A–E). Interestingly, the disease induced by the strains UA1114 and UA1511 was intermediate between UA946 and UA140, as determined by the size of the lesion and the severity score (Fig. 1A–E). When the parasitic loads at the site of infection (a parameter that reflects *in vivo* parasite multiplication) were determined, a striking correlation with the clinical behaviour was observed (Fig. 1F). Thus, the integration of the three parameters, namely lesion size, score and parasitic load, permitted to confirm that the four strains presented one of the following three patterns of virulence: high (UA946), moderate (UA1114 and UA1511) or low (UA140) (Table 1). An independent *in vivo* second experiment performed under similar conditions, revealed very similar results (Supplementary Fig. S1). Kruskal-Wallis test showed significant differences (p < 0,005) between strains behavior in size of the lesion and severity score data. Mann-Whitney test with Bonferroni corrections of p value to do pairwise comparisons showed significant differences between UA946 strain and the other three strains tested. Also, non-significant differences between the strains UA1114 and UA1511 for the two variables were observed. The parasite load was different between UA946 and UA140 (Supplementary Table 1).

**Table 1:** Virulence of the four strains of *L. panamensis* used in the present study according to the Mean (Median) ± SD values from the experimental variables.

Strain	Lesion intensity scale	Lesion area (mm^2^)	Parasitic load (parasites/ear)	Virulence (Inferred)
UA140	1, 0 (1) ± 0, 8	3, 2 (1, 8) ± 3, 6	4 × 10^10^ (4 × 10^5^) ± 8 × 10^10^	Low
UA1114	1, 8 (2) ± 0, 8	9, 5 (6, 9) ± 9, 8	9 × 10^10^ (7 × 10^10^) ± 8 × 10^10^	Moderate
UA1511	1, 9 (2) ± 0, 9	8, 7 (7, 7) ± 7, 5	8 × 10^10^ (7 × 10^10^) ±9 × 10^10^	Moderate
UA946	2, 6 (3) ± 1	15, 4 (12, 5) ± 12	2 × 10^11^ (2 × 10^11^) ±6 × 10^10^	High

This confirmed not only the value of the UA946 strain for genome sequencing, but also the sequencing of the other three strains for comparative genomics analysis.

### The genome of the virulent UA946 strain of *L. panamensis*

**Figure 2:**
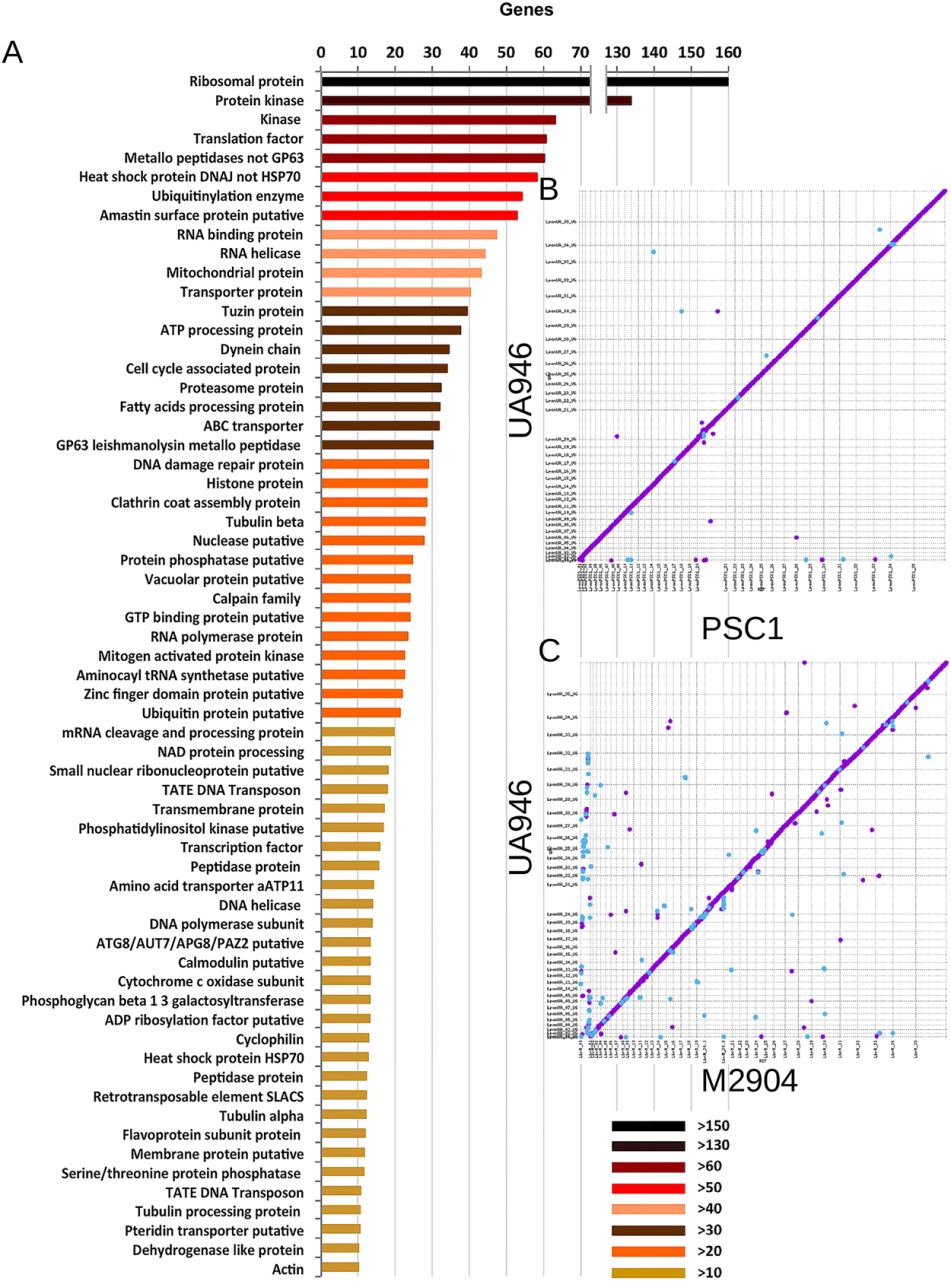
Functional annotation and synteny with *L. braziliensis* and *L. panamensis* genomes. (**A**) Distribution of ontologies of genes annotated in the UA946 assembly. (**B**) Structural comparison between the assemblies of the *L. panamensis* strains PSC-1 and UA946. (**C**) Structural comparison between the assembly of the *L. braziliensis* strain M2904 and the *L. panamensis* strain UA946.

Based on the evaluation shown above, the virulent *L. panamensis* UA946 strain was selected for genome sequencing and *de-novo* assembly, aiming to use it as reference for comparison to less virulent strains. Using a hybrid sequencing strategy we achieved a chromosome-level assembly for UA946 spanning 31,312,330 bp of genome size, distributed in 35 chromosomes. This is 2% larger than the genome of strain PSC-1 previously reported for the species (30,688,794 bp)^17^. Gene annotation revealed 8,094 gene models from which 8,034 showed high conservation relative to the annotated genes of *L. braziliensis* (M2904) and *L. panamensis* (PSC-1). The 60 new gene models have an average length of 642 bp, Codon Adaptation Index (CAI)^18^ of 0.68 (Supplementary Table S2), usage codon according to *L. braziliensis* genes and transcriptome mapping of amastigote and promastigote stage. One of the new gene models was located in a gap in the genome of strain PSC-1 and another two genes overlap a predicted deletion in the PSC-1 genome. However, these genes were not completely missing as indicated by the alignment of raw Illumina reads from PSC-1 to the gene models annotated in the UA946 assembly. One UA946 gene was partially deleted in PSC-1. Seven UA946 genes have sequences in PSC-1 without open reading frames (ORFs) and finally 49 genes have ORFs in PSC-1 without any annotation. 56% of the 8,094 genes correspond to “hypothetical protein, conserved” genes, 6% to “hypothetical protein, unknown function” genes, 1% to undefined genes and 37% to known genes (Fig. 2A). We also performed a reciprocal blast of the gene annotations to identify paralogous genes (Supplementary Table S3).

We performed pairwise synteny comparisons between the *L. panamensis* UA946 strain assembly and the assemblies of *L. braziliensis* M2904 and *L. panamensis* PSC-1 strains. Consistent with previous comparisons^17^, the three genomes were in general highly co-linear (Fig. 2B,C). As expected, the comparison between the assemblies of M2904 and UA946 strains revealed 365 potential structural variation events (SVs), whereas the comparison between the assemblies of *L. panamensis* strains only showed 111 potential SVs. However, half of the predicted SVs between M2904 and UA946 correspond to DNA that could not be placed in the correct contig segment of the M2904 assembly (First horizontal sequence in Fig. 2C).

We also aligned raw Illumina reads taken from the strains PSC-1 and M2904 to the UA946 assembly to look for confirmation signals of the structural events identified by the synteny analysis (See methods for details). As expected by the genetic distance between the strains, 80% of the PSC-1 reads and 71% of the *L. braziliensis* M2904 reads aligned to the UA946 genome. As a first attempt to find genes that could be related to virulence, we investigated insertions that could possibly include private UA946 genes and could be functionally related to virulence. Relative to the previously assembled genomes, we found 22 large (>200 bp) insertions in UA946 relative to PSC-1 confirmed by mapping (Supplementary Table S4). The two largest insertions (6,875 bp and 5,169 bp) located on chromosomes 19 and 29, respectively, completely cover two annotated genes: a COA ligase like protein and a tuzin. Most of the other insertions are shorter (less than 500 bp) and only one insertion partially spanned an annotated hypothetical protein. The same comparison procedure applied against the *L. braziliensis* strain M2904 revealed a larger number of 178 insertions (Supplementary Table S5, Supplementary File 1, Fig. 2). However, only 18 insertions completely covered annotated genes, 9 of them annotated as hypothetical proteins. Validation of the predicted insertions using alignments of short reads showed that none of the predicted insertions covering annotated genes shows the signatures of a novel DNA element. Hence, the insertions of complete genes identified by whole genome alignments were caused by copy number expansion of genes already present in the genome of M2904 and PSC-1 strains.

### Sequencing of *L. panamensis* strains with different levels of virulence

To compare the genomic variation among the different *L. panamensis* strains that are less virulent than UA946, Illumina sequencing was also performed on the strains UA140, UA1114 and UA1511. Raw reads obtained from each sample were independently aligned to the UA946 assembly. The alignment rates for these samples were in a range between 88 and 94% (Supplementary File 1, Fig. 3).

Based on Illumina read alignments to the UA946 assembly, we identified 656,782 variants, including copy number variants (CNVs, Supplementary File 2) and single nucleotide polymorphisms (SNPs, Supplementary File 3) in six strains including *L. panamensis* PSC-1 and *L. braziliensis* M2904 alignments for comparison in downstream analysis. These variants were distributed in 593,361 (90.34%) biallelic SNPs, 42,376 (6.45%) biallelic indels and 21,045 (3.21%) multiallelic variants. The high average read depth (>80x) of these 6 samples allowed to obtain accurate individual genotype calls for each variant. As expected, 88.6% of the SNPs and 79.4% of the indels correspond to differences interspecific between *L. braziliensis* and *L. panamensis* (Supplementary Table S6). Removing the *L. braziliensis* strain to identify polymorphisms within *L. panamensis* the total number of variants becomes 68,992 (10.5% of the total) distributed in 51,267 (74.31%) SNPs, 5,358 (7.77%) biallelic indels and 12,367 (17.92%) multiallelic variants. The percentage of biallelic SNPs located within protein coding regions (30% to 35%) is larger than the same percentage for other types of variants (4% to 5%). Including *L. braziliensis*, the total number of variants producing stop codons was 783 nonsense SNPs and 724 frameshift indels. Within *L. panamensis*, these types of mutations were reduced to 140 nonsense SNPs and 118 frameshift indels.

**Figure 3:**
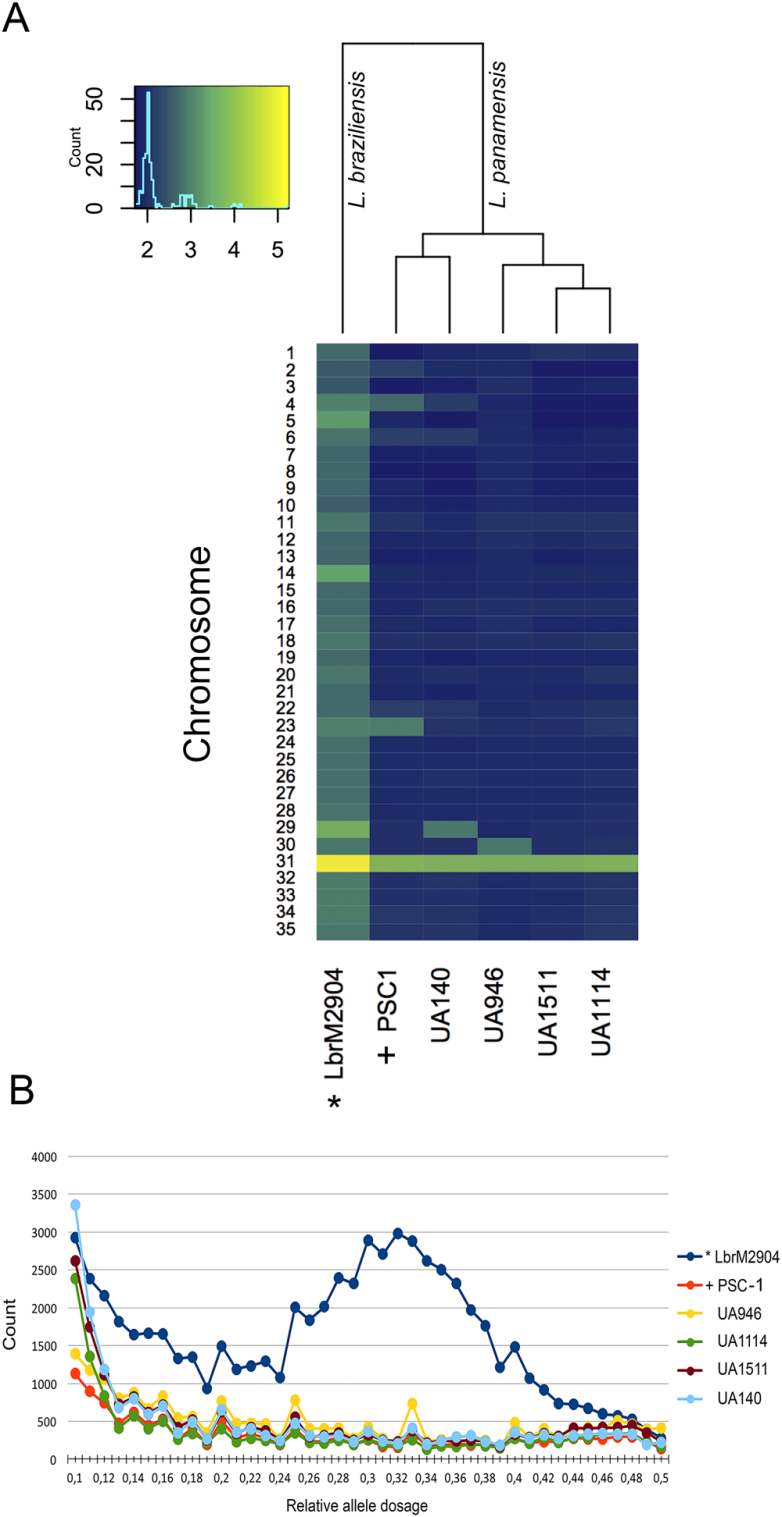
Comparison of ploidy between the four strains of *L. panamensis* with different virulence and reference genomes previously reported for the subgenus Viannia. (**A**) Normalized median read depth coverage using UA946 genome as reference. (**B**) Distribution of relative allele dosage among the six strains predicted from the relative allele counts at each site in the reference genome having base calls for at least two observed base pairs. *Reference genome of *L. braziliensis* reported^19^. ^+^Reference genome of PSC-1 reported^17^.

We estimated ploidy levels and compared them with the previously reported aneuploidies for the *L. braziliensis*^19^ and *L. panamensis*^17^ sequenced strains. Consistent with previous reports, this analysis shows that the five sequenced *L. panamensis* strains are predominantly diploid but the *L. braziliensis* strain is predominantly triploid (Fig. 3A). The overall distribution of relative allele frequencies (estimated from read counts) across the genome is clearly centred at 0.33 for the *L. braziliensis* strain M2904 (Fig. 3B). This indicates that the allele frequencies of heterozygous mutations should be predominantly in 2:1 proportions. For the *L. panamensis* strains, their allele distributions are flat with a small increase towards 0.5. This distribution combined with the low number of heterozygous SNPs identified for these strains indicates an overall ploidy of 2 with low levels of heterozygous. The main exception is chromosome 31, which shows a copy number of 4 for the 5 *L. panamensis* strains. Normalized average read depths per chromosome also show an increased ploidy in chromosomes of individual *L. panamensis* strains, including chromosome 23 of PSC-1, chromosome 29 of UA140, and chromosome 30 of UA946 (Fig. 3A).

### Analysis of Copy Number Variation (CNV)

**Figure 4:**
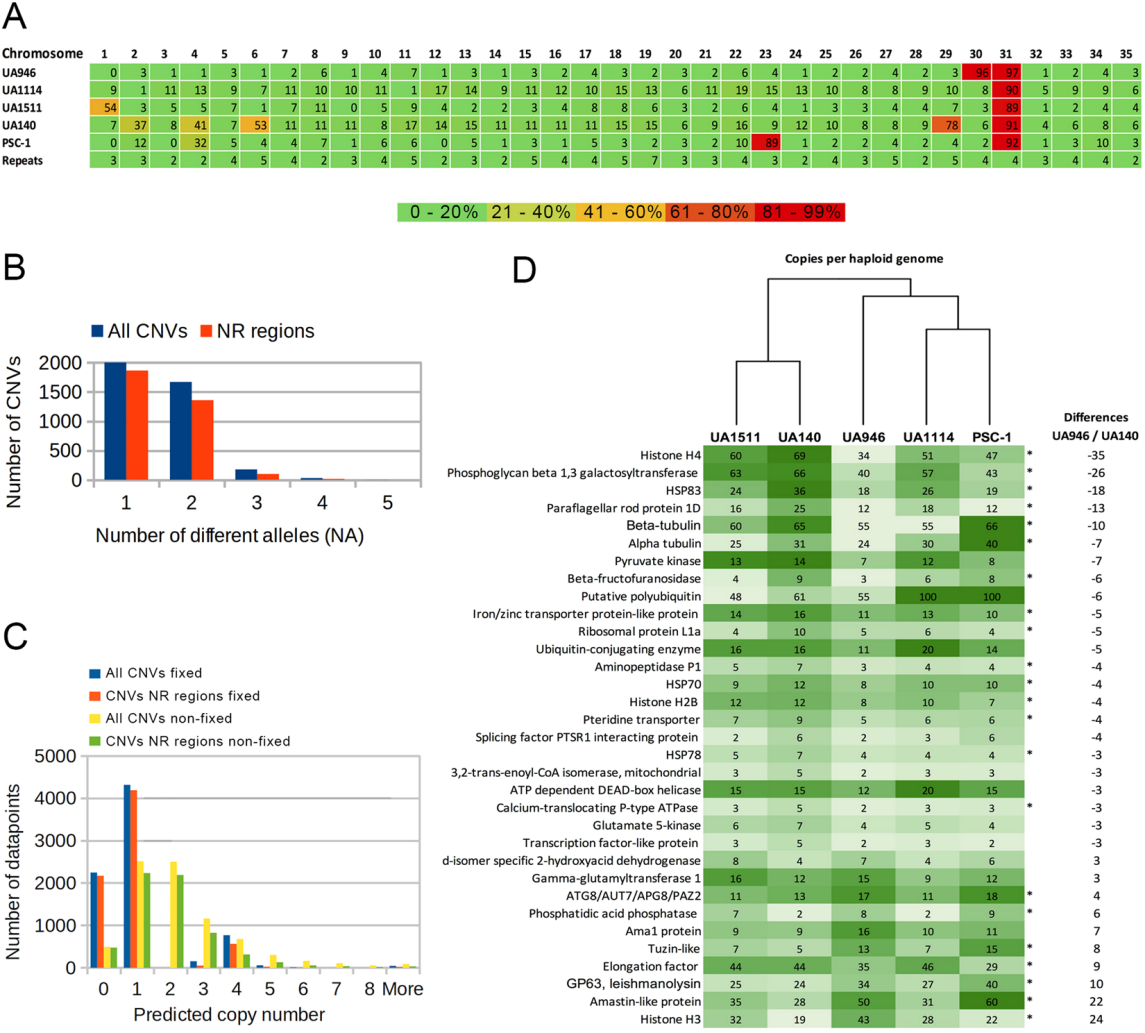
Analyses of duplications and copy number variation within *L. panamensis* genomes. (**A**) Percentage of each chromosome within each strain covered by predicted CNVs in which the copy number value (CN) is greater than 2 (e.g. duplications). (**B**) Distribution of CNVs by number of different observed CN values over the 5 strains for the complete CNV dataset and for the CNVs in non repetitive (NR) regions. (**C**) Distribution of predicted CN values on each strain for the complete CNVs dataset and of CNVs in non-repetitive (NR) regions. CNVs are classified by number of different CN values as fixed (only one value observed across the 5 samples) or non-fixed. (**D**) Differences in CN values among the 4 strains of *L. panamensis* evaluated with different virulence. *Genes involved in virulence or up-regulated in the amastigote state reported by different authors (Supplementary Table S1). The colours of the heatmap and the dendrogram were included with the purpose of highlighting the differences and similarity in CNVs among the strains analyzed. Reads of PSC-1 strain were included for comparison purposes^17^.

We performed a read depth analysis of the aligned data to identify regions of potential copy number variation (CNV) and predict the copy number (CN) for each gene within each *L. panamensis* sequenced strain (See methods for details). Considering that all *L. panamensis* strains look diploid, in this analysis the average read depth of each sample after correction for GC-content biases is related to the “normal” copy number (CN = 2) and significant local alterations of the average read depth are related to “abnormal” copy number. Combining the predictions for the five samples, we identified a set of 3,887 predicted CNVs spanning 12.6 Mbp of the genome (Supplementary Table S7). Consistent with the aneuploidies described above, predicted CNVs span more than 80% of the chromosomes 23, 29, 30 and 31, mainly because the span of duplications predicted separately for each strain reflects the copy number predicted from the relative read counts (Fig. 4A). In general, it is well known that the main source of false positive calls in any read depth analysis is the confounding effect of misalignments in repetitive regions of the genome. However, in this case, the assembly contains a very small portion of repetitive content for alignment purposes and hence 3,333 CNVs (85.8%) show less than 10% of intersection with repetitive elements.

Comparing the predictions of copy number within each CNV and within each strain, we identified 2,012 CNVs (51.8% of the total) showing a “fixed” abnormal copy number (CN ≠ 2) over the five samples. A total of 1,668 CNVs (42.9%) showed two different CN values across the five samples. The 207 (5.3%) remaining CNVs showed between three and five different CN values (Fig. 4B). The latter two categories are called “non-fixed” hereafter. The distribution of CN values predicted over the five strains and across all CNVs shows that CN = 1 (heterozygous deletion) was most common, followed by normal copy number calls (CN = 2) (Fig. 4C).

From the 3,333 CNVs in non-repetitive regions, 1,871 (54.7%) span annotated genes and furthermore 888 CNVs (26%) completely cover at least one gene. Considering only the 1,471 non-fixated CNVs in non-repetitive regions, 1,047 CNVs (71.2%) span annotated genes and 654 CNVs (44,5%) completely overlap at least one gene. This is encouraging to further investigate relationships between gene copy number variation and virulence. As expected, 403 of the latter cases are annotated as hypothetical proteins. Eight of these genes show differences ≥3 CN between UA946 and UA140. Considering the 94 genes previously related to virulence (Supplementary Table S1), whereas in general 548 (6.78%) genes seem to be duplicated within UA946 (either by aneuploidies or by copy number variation) according to the read depth analysis, 36 (37.5%) of the 94 virulence genes seem to be duplicated in UA946. Moreover, eleven of these genes showed differences ≥3 CN between the virulent UA946 strain and the less virulent UA140 strain. Functional annotations of these genes include heat shock protein, beta-tubulin, pteridine transporter, histones, peptidases, ATG8, phosphatidic acid phosphatase, tuzin protein and GP63 or leishmanolysin. Predictions of copy number for other genes possibly involved in virulence according to their functional annotations and having differences ≥3 CN between UA946 and UA140 are shown in Fig. 4D.

Finally, the analysis was also useful to test the presence of minichromosomes or circular episomes as reported in the previous assemblies^17,20^. Duplications were consistently reported for all *L. panamensis* strains on a segment of 37 kbp within chromosome 34 (1,351,000–1,388,000), which corresponds to about 70% of a previously reported minichromosome^17^ aligned by homology search with BLAST between 1,340,652 to 1,388,378. This region is interesting because it harbours approximately 13 genes involved in biological regulation, localization, response to stimulus, signalling and single-organism process according to gene ontologies. A larger minichromosome of close to 100 Kbp at the end of the same chromosome previously reported by Llanes *et al*.^17^ was consistently supported by two long duplications in PSC-1 spanning 80% of the region between 1,884,400 and 1,985,800, where the minichromosome is identified in the sequence. However, in contrast with the previous case, copy number estimation of the four strains UA sequenced in this study supports reference CN = 2 alleles for this region. Sampaio *et al*., showed that the presence of a similar mini-chromosome found in *L. braziliensis* favours the survival and infectivity^21^. Nevertheless, the copy number estimation for this region in the virulent strain UA946 suggests that the number of copies of this minichromosome is not related to the virulence level observed in this study.

### Gene diversity within *L. panamensis* and between *L. panamensis* and *L. braziliensis*

**Figure 5:**
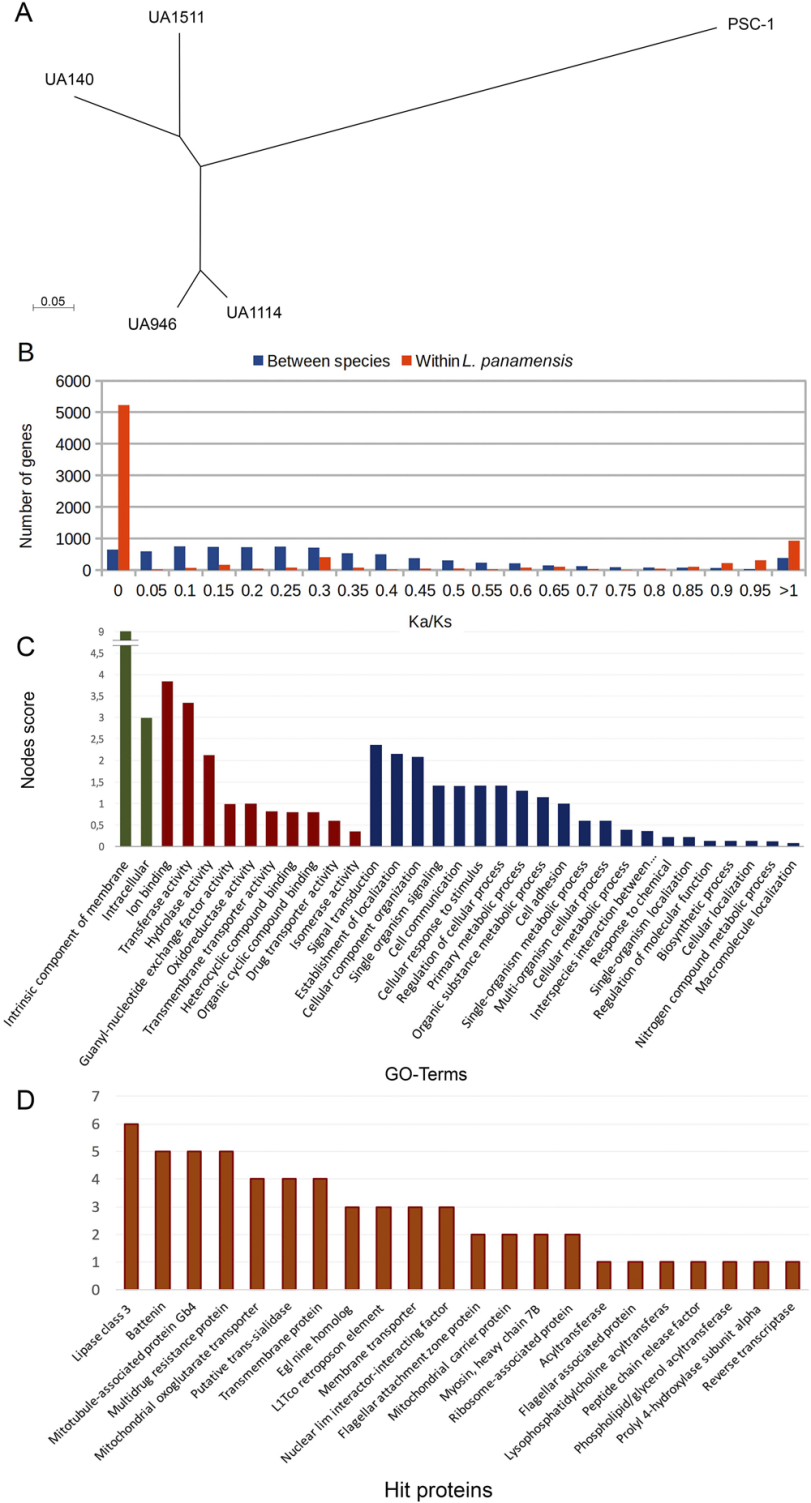
(**A**) Neighbor Joining dendrogram of *L. panamensis* strains based in Kimura 2-parameter distances (scale at bottom) expressed in units of number of base substitutions per site. The tree is drawn to scale with branch lengths, which is in the same units as those of the evolutionary distances inferred. (**B**) Distribution of Ka/Ks values estimated from SNPs in protein coding regions. Blue bars represent Ka/Ks values predicted from substitutions between *L. braziliensis* and *L. panamensis*. Red bars are Ka/Ks values predicted from polymorphisms within *L. panamensis*. (**C**) Gene ontology analysis at level 3 of the 81 hypothetical proteins possibly implicated in virulence according to machine learning techniques. Green: cellular component; red: molecular function; and blue, biological process. (**D**) Proteins identified in the annotation of the 81 hypothetical proteins that are part of 230 possible new genes associated to virulence (Blast2GO program).

Using a filtered dataset of 14,793 SNPs genotyped on the five *L. panamensis* strains after removing repetitive regions or CNV regions for at least one strain, we built a neighbour-joining dendrogram to compare SNP based predicted genetic distances between the strains (Fig. 5A). The strain PSC-1 is clearly separated from the UA strains sequenced in this study. UA946 clusters together with strain UA1114 whereas the less virulent strains UA140 and UA1511 appear more separated. In contrast to the number of heterozygous variants, ranging from 2,000 to 3,000 for the five strains, the number of homozygous differences with UA946 is more than 2,500 for UA140 and UA1511 and grows to 7,653 for PSC-1.

Because the number of samples to assess variation in virulence is limited, it is generally difficult to identify associations between genetic variants and virulence levels using conventional techniques such as Genome-Wide Association Studies (GWAS) or Bulk Segregant Analysis (BSA). Hence, we investigated the patterns of protein allelic diversity between and within species that could be inferred from SNPs using the Ka/Ks ratio (also known as dN/dS) as a standardized measure^22,23^ and investigated if these patterns could provide information about genes related to virulence.

We estimated Ka/Ks values separately from SNPs differentiating *L. braziliensis* and *L. panamensis* (between species, also called substitutions) and from the SNPs identified only within *L. panamensis* (within species, also called polymorphisms). As expected based on the overall number of SNPs, Ka/Ks values between species showed an average of 0.36 calculated over the 7,854 genes with at least one synonymous mutation whilst within *L. panamensis* was 0.27, calculated over only 2,779 genes with at least one synonymous mutation. Figure 5B shows the distribution of Ka/Ks values in both datasets. Consistent with the distribution of SNPs, Ka/Ks values indicate that the observed variability between species is much larger than the variability within *L. panamensis*. Interestingly, the last four bars of the histogram seem to show an unexpectedly large number of genes with high Ka/Ks values within *L. panamensis*. A closer look to these categories revealed that they were mostly composed by 1,417 genes having zero synonymous mutations and a moderate number (1.43 on average) of non-synonymous mutations. The same pattern of variation was observed between species, but only in 118 genes, and with an average of 2.63 non-synonymous mutations.

Ka/Ks has been proposed as a statistic to test selection in protein evolution. Informally, under a Wright-Fisher model without selection, mutations in synonymous and non-synonymous sites should appear at a similar rate and hence the Ka/Ks ratio should be close to 1^23^. Whereas values significantly smaller than 1 could indicate purifying selection, values larger than 1 could indicate positive selection. However, recent simulation studies show that this test should only be applied to substitutions between species because the assumption of sampling from divergent lineages is violated for polymorphisms within species^24^. Because other tests of intraspecies selection require large sample sizes, we only looked for signatures of positive selection from the SNPs differentiating *L. braziliensis* and *L. panamensis*. Only 7 genes showed Ka/Ks ratios significantly larger than 1, none of them previously related to virulence. Three of these genes were annotated respectively as a viscerotropic leishmaniasis antigen, an argininosuccinate synthase and a cysteine peptidase.

Taking into account the large overall amount of observed variability between species compared to that within species, we also investigated genes in which higher Ka/Ks values are observed within *L. panamensis* than between species. Whereas in general, these were only 2,188 (26.9%) genes, within the genes related to virulence, 38 (39.6%) present the same pattern of variability. This suggests that non-synonymous point mutations within virulence genes can be drivers of the observed variability in virulence within the sequenced strains. High Ka/Ks values were observed in virulence genes involved in intracellular transport, autophagy and cell remodelling, pteridine transport and gene expression. Whereas 36 virulence factors only showed variability between species, six surface protein genes were variable only within *L. panamensis* (Supplementary Table S8).

### Machine learning to predict virulence genes from genomic variation of limited samples

Although Ka/Ks values within *L. panamensis* and between species, as well as predicted values of copy number showed interesting patterns within the genes related to virulence, none of the Ka/Ks ratios or estimations or copy number can be used independently as good predictors to completely distinguish virulence genes. Other annotated genes may be involved in virulence or may be important in the amastigote state. Moreover, more than 60% of the *L. panamensis* genes products are annotated as “hypothetical proteins”. However, the observed partial associations are encouraging to try to identify novel candidate genes for virulence using a supervised learning approach able to combine the partial information that seems to be provided by the different analyses of genomic variability. Hence, we tried several machine learning approaches to perform automated identification of virulence genes using as features information of gene diversity between and within species (non-synonymous mutations and Ka/Ks values), as well as predictions of copy number and heterozygous within individual strains (see Methods for details). Genes not previously related to virulence were tested against 200 models built running commonly used machine learning techniques (Support Vector Machines, Naive Bayes and Random forests) using different random subsets of genes as negative cases. As expected, each experiment predicted association with virulence for a different set of genes. Whereas the random forest approach consistently predicted relation to virulence for the largest number of genes (1113 on average), the support vector machine predicted virulence for the lowest number of genes (97 on average). Within each prediction model, almost all genes predicted by the support vector machine and over 80% of the genes predicted by naive Bayesian approaches were predicted by at least one additional method (Supplementary File 1, Fig. 4, Supplementary Table S9). Taking the union of all models, 230 genes (2.88%) were constantly associated to virulence in 100 or more models. 59 of these were homologous to previous list of 94 genes reported to be involved in virulence or up-regulated in amastigote stage and 80 genes were annotated as hypothetical proteins are predicted as new genes involved in virulence and represent new potential targets for antileishmania drug design. However, a homology search with BLAST itself found 14 genes duplicated which may not be good targets for drug design. An ontology analysis of 66 remaining genes using the Blast2GO tool^25^, showed the biological processes and molecular function in which these genes are involved. Most of these processes occur at the membrane level (Fig. 5C). Similarly, the annotation revealed by Blast2GO showed 18 proteins with diverse functions (Fig. 5D). Additionally, the use of the TargetP, SignalP and TMHMM programs showed that within the 66 possible new proteins involved in virulence, 14 are possibly located in the kinetoplast, 8 contain signal peptide, 4 have transmembrane helices, and two of them more than 10 helices (Supplementary Table S10).

## Discussion

The virulence in leishmaniasis is not only the result of the genotypic characteristics of the strains, but it is also the result of the response of vertebrate host^14,26,27^. In the present study, all the parameters of experimental inoculation in mice were controlled. BALB/c mice were used, which are considered a susceptible strain to typical lesions of CL^28^, and a constant amount of inoculum of 10^5^ promastigotes was used for all the experiments. A previous study in *L. major* showed that inoculations of 10^5^–10^7^ parasites produce large lesions in BALB/c mice unlike inoculations with fewer parasites^29^. Ear (intradermal) was also chosen as the site of inoculum. This site has been previously reported as capable to generate Th2 immune response, leading to the development of persistent lesions in infections with *L. major*^30^. However, the mosaicism^31^, constitutes a major methodological problem for the analysis of structural variation genomics in Leishmania. Prieto Barja *et al*.^32^, found that *L. donovani* is more aneuploid *in vitro* culture than in vertebrate host, establishing trisomy in chromosomes 5, 9, 23 and 26 *in vitro* passages. It is possible that the number of subpopulations of karyotypically different parasites *in vitro* culture increases. Likewise, there are fluctuations in allele frequencies during the *in vitro* culture, making it difficult to interpret the chromosome somy based on allele frequency. Therefore, it is important to perform DNA extraction in each passage. In this work all strains, before being subjected to whole genome sequencing, were isolated from the ear of the infected mice and grown *in vitro* by the same short period of time in order to minimize the effect of aneuploid variation on the analysis. In this regard, the experimental variables that could affect the results, different from the characteristics of the strains used, were controlled to the maximum. Previous studies have shown a relationship between the genotypic variation in strains of *L. major* and heterogeneity in size of the lesion and immune response generated in BALB/c mice under controlled conditions^33–35^.

This work is the first attempt to assess the role of genomics variation in virulence within the subgenus Viannia. Analysis of CNVs between the strain with increased virulence (UA946) and the strain with low virulence (UA140) showed 22 genes with variation in CN involved in survival and replication of amastigotes (Supplementary Table 1, Fig. 4D). Several studies had shown how the overexpression or depletion of some of them directly affect the virulence in BALB/c mice and cellular remodelling and differentiation, such as GP63^36–40^, glycoconjugates and GPI-anchored proteins secreted and/or expressed on the surface^41,42^, Beta 1,3 galactosyltransferase^43–45^, Phosphatidic acid phosphatase proteins^46–48^, amastin surface proteins^49^, biopterin transporter^50–54^, ATG8^55–60^, and histone proteins^61–63^.

Whole-genome sequencing (WGS) studies in *L. donovani* strains from two regions in Ethiopia also found CNVs in genes involved in virulence or up-regulated in amastigote stage (Folate/biopterin transporter, Hydrophilic acylated surface protein, Mannosyltransferase, Amastin-like protein)^64^. Dumetz *et al*., demonstrated that Leishmania has the ability to pre-adapt to different stress conditions^65^. Strains of *L. donovani* studied have an intrachromosomal amplification of genes involved in resistance to pentavalent antimonials (Sb) that allow them to survive to the direct exposure to the maximum concentration of the drug. In this amplified fragment, in addition to genes involved in redox pathways and drug resistance, genes associated with virulence were also found (Hydrophilic acylated surface and Membrane-bound acid phosphatase proteins). The *L. panamensis* UA946 strain, sequenced in the present work, was isolated more than 15 years ago from a patient with cutaneous leishmaniasis and maintained *in vivo* passages in BALB/c mice. Hence, UA946 strain could have undergone a process of selection that favors infection in BALB/c. This would be a desired scenario for future studies of pathogenesis and could explain the results shown in this work.

One of the main factors thought to affect gene dosage is the chromosome copy number variation, which makes it possible to find copies of extra chromosomes due to the extensive aneuploidy confirmed at the population level in the genus *Leishmania*^19,20^. In this study, especially on chromosomes 29–31, a relationship between the ploidy variation shown in Fig. 3 and the CNVs data shown in Fig. 4 is obvious, with no apparent repeats bias (Fig. 4A). Downing, evaluating seventeen *L. donovani* isolates with sensitive and resistant phenotypes to pentavalent antimonial (SSG), did not find significant association between the observed aneuploidy and SSG resistant phenotype^20^. However, Dumetz, evaluating the modulation of aneuploidy as a primary strategy to adapt to drug pressures in *L. donovani*, found a direct association between the chromosomes copy numbers, dosage and expression of specific genes^66^. Moreover, evidence of intrachromosomal amplification as mechanism to generate CNVs in New World Leishmania has also been reported en *L. amazonensis*^67^ and in the Viannia subgenus in *L. guyanensis*^68^ and in the *L. braziliensis* – *L. peruviana* complex^69^.

The highly conserved protein core (7157), common among *L. mexicana, L. infantum, L. major, L. braziliensis* and *L. panamensis*^17^, the few species-specific genes reported in studies of comparative genomics analysis^17,19,70–72^, and the results shown here, suggest that the virulence is probably influenced by differences in gene expression and dosage of this common conserved protein core in *Leishmania spp*. Different authors suggest that, in the absence of transcriptional control in *Leishmania*, it is possible that mechanisms such as amplification^20,68,73–75^, gene duplication^76^ or modulation of aneuploidy^66^ have evolved as mechanisms for altering mRNA levels, generating an extensive phenotypic heterogeneity that is subjected to selective forces such as drug resistance^20,73,77,78^, or as shown here, subjected to adaptation to animal models. In infections in hamster *L. donovani* is more disomic in parasites that infect the liver compared to the spleen, demonstrating that aneuploidy variation is also dependent of infected tissue^32^. Similarly, clones of *L. donovani* from Ethiopian patients with HIV co-infection isolated from the skin and spleen of the same patient showed different karyotypes^64^. Aneuploidy equally affects the gene dosage and the selection of beneficial alleles against different environmental changes.

Studies in *L. donovani*, found SNPs associated with drug resistance, with allelic frequencies that increased progressively with the concentration of Potassium Antimony Tartrate (PAT), evidencing selection of genotypes under pressure^65^. Assessing virulence in Viannia subgenus in an experimental setting is a difficult task that could only be done for a small number of strains. Analysis of protein evolution through the Ka/Ks statistic has been useful in other studies to assess positive selection in genes associated with virulence in *Trypanosoma*^79^, *Plasmodium*^80^, *Fasciola*^81^, Zika^82^, among others. The Ka/Ks analysis reveals that genes implicated in virulence such as ADP ribosylation factor, amastin like protein, aminopeptidase, ATG8, cysteine peptidases, elongation factors, folate/biopterin transporter, GP63, histones, HSPs, ppg3, among others, show allelic variability in *L. panamensis* even within the low number of sequenced samples (Supplementary Table S8). Thus, genomic adaptation strategies such as amplification of gene copy number or protein evolution through single nucleotide mutations can be a response to pressures in *Leishmania* spp. Further analyses with larger intraspecies genetic variability and other models could assess if the protein evolution is guided by positive selection to confer resistance to the host immune system. From a statistical perspective, these relatively orthologous sources of partial association to virulence could be interpreted as features and combined using one of the several well known machine learning techniques to try to build a model that allows to prioritize genes for further functional association studies (Supplementary Table S9). Following this approach, we predicted 230 genes as novel candidates for relation to virulence. However, some genes homologous to virulence factors (Supplementary Table S1) were included in the output dataset predicted by different machine learning approach (ribosomal proteins, amastin surface proteins, tubulins, elongation factors, folate/biopterin transporters, heat shock proteins, histones, kinesins, metallo-peptidases, paraflagellar rod protein, phosphoglycan beta 1,3 galactosyltransferases, proteophosphoglycans, surface antigen protein, tryparedoxin peroxidase, tuzin proteins, among others), showing the efficiency of the predictions. Of the 230 genes, 81 were annotated as hypothetical proteins. Being about 60% of the genes in *Leishmania* annotated as a hypothetical, the results shown here propose new targets to be prioritized for evaluation in the search for novel antiparasite therapies. Similarly, the present study shows how the machine learning strategies supported in biological traits generate data applicable to parasitological studies. Moreover, the annotation made to the new 81 hypothetical proteins possibly implicated in virulence demonstrates the need to review the annotation of the current *Leishmania* genomes.

Finally, this study highlights the importance of deep sequencing and genomic structural variation analyses in exploring the virulence of *L. panamensis* strains. Using a combination of studies *in vivo*, bioinformatics analyses and machine learning, we provide valuable insights indicating that the virulence of *L. panamensis* could be studied by the CNVs and SNPs. Different studies have shown that the subgenus Viannia species are similar in their genetic variability^83^ and genomes^84^. Thus, the findings shown here could also be applicable to others species of panamensis/guyanensis/shawi cluster. Studies evaluating the progressive genomic changes that mediate the adaptation of *L. panamensis* to BALB/c mice, that measure the CNVs and the allelic frequencies of SNPs during the course of the infection, and its comparison with previously reported in other species are necessary to contribute to the study of genomic instability as a possible mechanism that favours the adaptation to tissues and tropism, the susceptibility to drugs and virulence or degree of pathogenicity.

Our data provide the baseline to understanding the virulence of *L. panamensis* strains, and future studies will require a validation with a higher number of strains and functional genomic approaches are expected to complement the results shown here, for a better understanding of the mechanisms that control infections with different virulence.

## Methods

### Animals, parasites and DNA preparation

Female, 6–10 weeks old BALB/c mice (Charles River, USA) were maintained in a SPF animal facility at the Sede de investigación universitaria (SIU), Universidad de Antioquia. Ethical approval for all *in vivo* procedures was obtained by the Animal Ethics Committee of the Universidad de Antioquia, Colombia. All animals were handled in strict accordance with good animal practice as defined by the Colombian Code of practice for the care and use of animals for scientific purposes, established by Law 84 of 1989. Four *L. panamensis* strains, coded as UA140, UA946, UA1114 and UA1511, isolated at the “Programa de estudio y control de enfermedades tropicales, PECET (Universidad de Antioquia, Medellin, Colombia)” from patients suffering ACL, were used in this study. The UA140 strain is routinely kept by long term *in vitro* passages with occasional *in vivo* passages, and it is essentially avirulent in BALB/c, since no or very small self-limited lesions are typically observed in most of mice after infection. The UA946 strain has been adapted to grow in BALB/c mice for years and reproducibly induces large cutaneous ulcerative lesions with eventual necrosis and mutilation in infected mice. The UA1114 and UA1511 isolates have been kept *in vitro* cultures and used to infect BALB/c mice in several rounds of serial infections (3–5 *in vivo* passages). These two strains induce cutaneous lesions in a less reproducible manner in BALB/c mice, with variable amounts of mice exhibiting nodular or ulcerative lesions. The species identity of the four isolates was confirmed by mAb and isoenzymes. Promastigotes were grown at 26 °C in NNN or Schneider’s Drosophila medium (Sigma, USA) supplemented with 10% heat-inactivated FCS and 2% filtered sterile human urine. Five to six-day cultures (early stationary phase) were used for all purposes. The DNA of the four strains was extracted from 10^9^ promastigotes harvested in early stat phase of growth, using the DNEasy DNA Purification Kit (Qiagen). DNA quantity and quality was assessed by Nanodrop.

### Infections, follow up and parasite burden determination

5–7 animals per group were infected into the right ear (id) with 10^5^ stationary promastigotes (in 20 uL sterile PBS). The measurement of the size of the lesion was made calculating the diameter of the lesion, provided an accurate and representative measurement of growth of the lesions during the course of the infection^85–87^. Lesion measurements were performed weekly, by registering the two crossed diameters of lesions, and calculating the lesion area (in mm^2^) with the formula $A=\pi {(\frac{D1+D2}{4})}^{2}$. As a complementary clinical follow up, a severity “Score” (scaling from 0 to 4) was also implemented based on the appearance of the lesion, as follows: Score 0: no apparent lesion. Score 1: ear with small nodular lesion with no clear ulceration. Score 2: large nodular lesion or small nodular lesion that begins to ulcerate. Score 3: Frank ulcer. Score 4: large ulcers with necrotic areas and/or mutilation. For parasitic loads determination, mice were sacrificed at the 8^th^ week post-infection and the infected ears removed to be utilized to quantify the amount of viable parasites by using a limiting dilution assay^88^. Clinical (size of the lesion and Score) and parasitological (number of viable parasites per ear) parameters were recorded in individual mice, and the mean, median and +/− SD were calculated and compared among groups. Promastigote cultures were also prepared from the ears of UA946-infected mice to be used for DNA preparation and genome sequencing by 454 technology. In a second *in vivo* experiment, similar samples were obtained from UA946-, UA140-, UA1114- and UA1511-infected ears for genome sequencing using Illumina technology.

### Genome sequencing and assembly of the high virulent UA946 *L. panamensis* strain

Genome sequencing of the UA946 strain for *de-novo* assembly was performed on a combination of two different protocols for whole genome sequencing (WGS): paired end 454 GS FLX titanium Shotgun (8 kbp insert size, mean read length 450 bp), and paired end Illumina HiSeq (350 bp insert size, 100 bp read length), SRA accession SRP154327, BioProject: PRJNA481617. The quality of the reads was assessed by FastQC (http://www.bioinformatics.babraham.ac.uk/projects/fastqc/), and using PRINSEQ program^89^, 6% of the reads having an average Phred quality score lower than 30 were removed. *De novo* assembly was carried out with the program NEWBLER v2.9^90^. Sequencing data generated for UA946 strain genome assembly represented an expected 22-fold median coverage assuming a genome size of 31 Mbp. *De-novo* assembly produced a high quality genome with: 90 scaffolds, 31.2 Mb of total length and N50 of 620 kb (average length 345 kb). The maximum length of a scaffold was 1,534,379 bp. We also performed Illumina whole genome resequencing for UA946 (paired-end read 2 × 101 bp). 91.62% of these reads were aligned to the 454 assembly, and these were used to iteratively correct errors in the consensus sequence by iteratively mapping reads to the sequences using iCORN^91^ and close gaps using IMAGE^92^ through 24 iterations with different k-mers. ICORN corrected 1847 base errors and indels in 12 iterations and from the 1,130 gaps (1,298,188 bp) initially observed in the 454 assembly, the IMAGE closed 806 gaps (150,552 bp) and obtaining a final genome size of 31,390,823 bp (Supplementary File 1. Table 2).

Sixty-six of the 90 assembled scaffolds (31′312,814 bp) were oriented and assigned to the 35 chromosomes using MUMmer v3.23^93^ and ABACAS^94^ programs and confirmed by 25 PCR reactions of adjacent scaffolds. Additionally, through BLASTn itself, ACT^95^ and orientation of paired end reads, the orientation of contigs within the scaffolds was verified. Per-base quality of the assembly was evaluated using the software bowtie2 v2.2.3^96^, and QualStats and CoverageStats commands of NGSEP v3.1.0^97^. Even at 3′ end of reads, the percentage of different bases with respect to the genome assembly does not exceed 1% and 2% in 454 and Illumina reads, respectively (Supplementary File 1, Fig. 5A,B). Alignments of 454 reads achieved a 21-fold median coverage and Illumina reads achieved a 112-fold median coverage (Supplementary File 1. Fig. 5C,D). Analysis of paired-end reads that were not properly aligned in pair, and PCR assays to identify potential misassembles revealed only three cases that were manually corrected. A complete summary of the genomic characteristics of the strain UA946 of *L. panamensis* and its comparison with other species of *Leishmania* spp., is shown in Supplementary File 1. Table 3. The other three strains with reduced virulence were sequenced by Illumina protocols for HiSeq 2 × 101 bp paired-end reads with 350 bp insert length achieving a raw average read depth per sample larger than 85x.

### Identification of repetitive regions for read alignment in the UA946 assembly

We performed two separate analyses to identify regions that can be considered as repetitive for short read alignment purposes: a self homology search with BLAST for repetitive regions of at least 500 bp and the clustering algorithm implemented in NGSEP based on multiple alignments of short Illumina reads^97^. Predictions in the first dataset were largely (97.4%) contained in the second dataset. While the first method reported 839 Kbp of repetitive sequence (2.7% of the total genome size), the second method reported 1.16 Mbp of repetitive sequence (3.7% of the total). Low percentages (<8%) of repetitive content were observed across the 35 mapped chromosomes and a high percentage (almost 50%) of repetitive content was only observed in the 78 Kbp of sequence not assigned to a chromosome (Supplementary File 1. Fig. 6).

### Genome gene annotation of a high virulence *L. panamensis* strain

The annotated *L. braziliensis* (M2904)^70^ and *L. panamensis* (PSC-1)^17^ reference genomes were transferred to the assembled virulent *L. panamensis* UA946 draft genome based on sequence conservation and synteny with RATT^98^. Gene structure and functional annotation were manually inspected and edited using the Artemis program^99^. To improve the *L. panamensis* genome, new gene models were identified by using a combination of CodonUsage, Codon Adaptation Index (CAI)^18^, BLASTx and transcriptome mapping of promastigotes/amastigotes reads (454 shotgun protocol) on Open Reading Frames (ORFs) exceeding 200 bp in length. Several programs were used for functional annotation of the new gene models. We used the InterPro scan application of Blast2GO^25^ to search protein domains. TargetP 1.1^100^, SecretomeP 2.0^101^, SignalP 4.1^102^, TMHMM 2.0^103^, were used to evaluate the cellular localization of the proteins inferred from the annotation process and if these proteins are secreted or are transmembrane.

### Whole genome comparison between the genomes of UA946, PSC-1 and M2904

Whole genome comparisons between the assemblies of the *L. panamensis* strains UA946, PSC-1 and between UA946 and the assembly of the *L. braziliensis* strain M2904 were performed using the package Mummer^97^. To validate the structural events, in particular the insertions within UA946, predicted by the whole genome alignments described above, we aligned to the UA946 assembly publicly available Illumina reads of M2904 and PSC-1, as well as the Illumina reads of UA946 sequenced for this study. Then, the average read depth within the predicted insertions with the average read depth across the genome was compared. Real DNA insertions in one strain relative to another can either be caused by new DNA segments only present in one assembly or by relocations of mobile or repetitive elements. Only the first case should be supported by a significant reduction in read depth for an alignment of reads taken from the strains not having the new DNA segment. In contrast, insertions of mobile repetitive elements should not produce a reduction of read depth within the region. In both cases, read pairs aligning at a distance significantly larger than the library average insert length should flank real insertions. Supplementary File 1. Fig. 7 shows an example of a 1 Kbp insertion within UA946 for which zero coverage and a large number of read pairs with abnormally large predicted insert length are observed flanking the region with the predicted insertion.

### Read alignment and variants identification

Reference guided analysis of the Illumina data for each of the six samples included in this study was performed with the NGSEP pipeline v3.1.0^97^.Reads for each sample were aligned to the UA946 assembly using bowtie2 v2.2.3^96^,with default parameters for paired-end reads except for the maximum number of alignments to keep for each read (-k parameter), which was set to 3. Alignments were sorted by reference coordinates using picard (https://broadinstitute.github.io/picard/). Plots of differences against the reference genome per read position and read depth distribution were obtained running the commands QualStats and CoverageStats of NGSEP. The FindVariants command of NGSEP was then executed for each sample with the recommended parameters for Illumina WGS data: 1) Minimum genotype quality 40; 2) Minimum value allowed for a base quality score 30; and 3) Maximum number of 2 alignments allowed to start at the same reference site. Merging of variants from the 6 samples were performed following the recommendations available in the NGSEP documentation, including the command MergeVariants to obtain the set of variants across the samples, the command FindVariants to genotype the variants obtained in the previous step on each sample and the command MergeVCF to assemble the final dataset of variants genotyped in the six samples in variant call format (VCF). The command FindVariants also produce for each sample the calls of Copy Number Variation (CNVs) described in the results section. Merging of these calls into a consolidated set of 3,978 regions affected by CNVs was performed using the same heuristic clustering and genotyping procedure described for characterization of CNVs in common bean^104^.

The NGSEP commands Annotate, FilterVCF, and VCFDistanceMatrixCalculator were used respectively to perform functional annotation of variants, filtering and construction of distance matrices. The un-rooted dendrogram shown in Fig. 5 was built using the neighbour joining algorithm from a distance matrix calculated from a filtered VCF file having SNPs within *L. panamensis* in non-repetitive regions of the genome, and genotyped in the 5 *L. panamensis* strains. These filters were enforced using the following options of the FilterVCF command of NGSEP: “-saf” to provide the ids of the *L. panamensis* strains, “-fi” to filter invariant sites within *L. panamensis*, “-minI” to keep variants genotyped in the 5 strains, and “-frs” to remove repetitive regions. The Annotate and VCFDistanceMatrixCalculatorCommands were executed with default parameters.

Counts of synonymous and non-synonymous sites per gene and Ka/Ks values were estimated directly from the VCF file and the reference genome following an approximate approach implemented in a custom script. In brief, for each position of each codon, point mutations are simulated and counted as synonymous if the corresponding aminoacid does not change, or non-synonymous if the corresponding aminoacid changes. All changes were considered equally probable, so this does not account for transition/transversion ratio or for codon usage.

### Machine learning model to predict Virulence genes

Based on literature review, we selected a set of 94 genes, which were validated as known virulence genes or implicated in amastigote stage (Supplementary Table S1). The other genes were distributed in 50 random groups of 160 genes per group. Then, we built a total of 450 different binary classification models to infer genes related to virulence using the software tool Weka^105^. Each model is built using one of the 50 random groups as a negative dataset for virulence and running one of the following machine learning approaches implemented in Weka: IBk, a J48 tree, K-Star, default naive Bayes (NBDef), naive Bayes with a kernel density estimator for numerical values (NBKernel), Random Forest (RF), the default support vector machine (SVMDef), a support vector machine with conjugate gradient descent (SVMConjugate) and the ZeroR method which is recommended as a lower bound for the other methods. Selected features for each gene include the number of non-synonymous substitutions and the estimated Ka/Ks between *L. braziliensis* and *L. panamensis* and the Ka/Ks ratio estimated from synonymous and non-synonymous substitutions, the number of non-synonymous polymorphisms within *L. panamensis*, the Ka/Ks ratio estimated from synonymous and non-synonymous polymorphisms, the raw prediction of copy number for UA946, UA1114, UA140 and UA1511 and also the number of heterozygous SNPs within the same four strains (12 features in total). Cross validation was performed in each model to assess its the predictive accuracy. Both SVM methods yielded identical results and were the most conservative across models with low false positive rate (below 0.05) but also low true positive rate (0.14 on average). The naive Bayesian approaches showed a larger but still small false positive rate (below 0.05) compared to the SVM, increasing the true positive rate over 0.25. The random forest approach increased the true positive rate to 0.42 but also increased the false positive rate to 0.16. Because the other methods do not seem to provide further improvement over the random forest, we decided to perform prediction of virulence considering only the models built using the SVM, the two Naive Bayesian approaches and the random forest (200 models). The complete set of genes obtained after excluding the 94 genes related to virulence was provided to Weka for discovery of new genes related to virulence using each model. A gene was predicted as related to virulence if at least 101 of the 200 models classify the gene as related to virulence. This corresponds to an ensemble model in which the decision on classification is taken by majority vote. The number of models that classify each gene as related to virulence is reported in Supplementary Table S9.

## Electronic supplementary material

Dataset 1

Dataset 2

Dataset 3

Dataset 4

Dataset 5

Dataset 6

Dataset 7

Dataset 8

Dataset 9

Dataset 10

Dataset 11

Dataset 12

Dataset 13
