# Supplementary material for: Genomic Analysis of Colombian Leishmania panamensis strains with different level of virulence
Source: Sci Rep. 2018 Nov 26;8:17336. doi: 10.1038/s41598-018-35778-6 (PMC6255768; doi:10.1038/s41598-018-35778-6)
Supplement: Supplementary file 1 — Dataset 1 [file 41598_2018_35778_MOESM1_ESM.pdf]

Genomic Analysis of Colombian Leishmania panamensis strains with different level of virulence

Daniel Alfonso Urrea, Jorge Duitama, Hideo Imamura, Juan F. Álzate, Juanita Gil, Natalia Muñoz, Janny Alexander Villa, Jean-Claude Dujardin, José R. Ramirez-Pineda, Omar Triana-Chavez

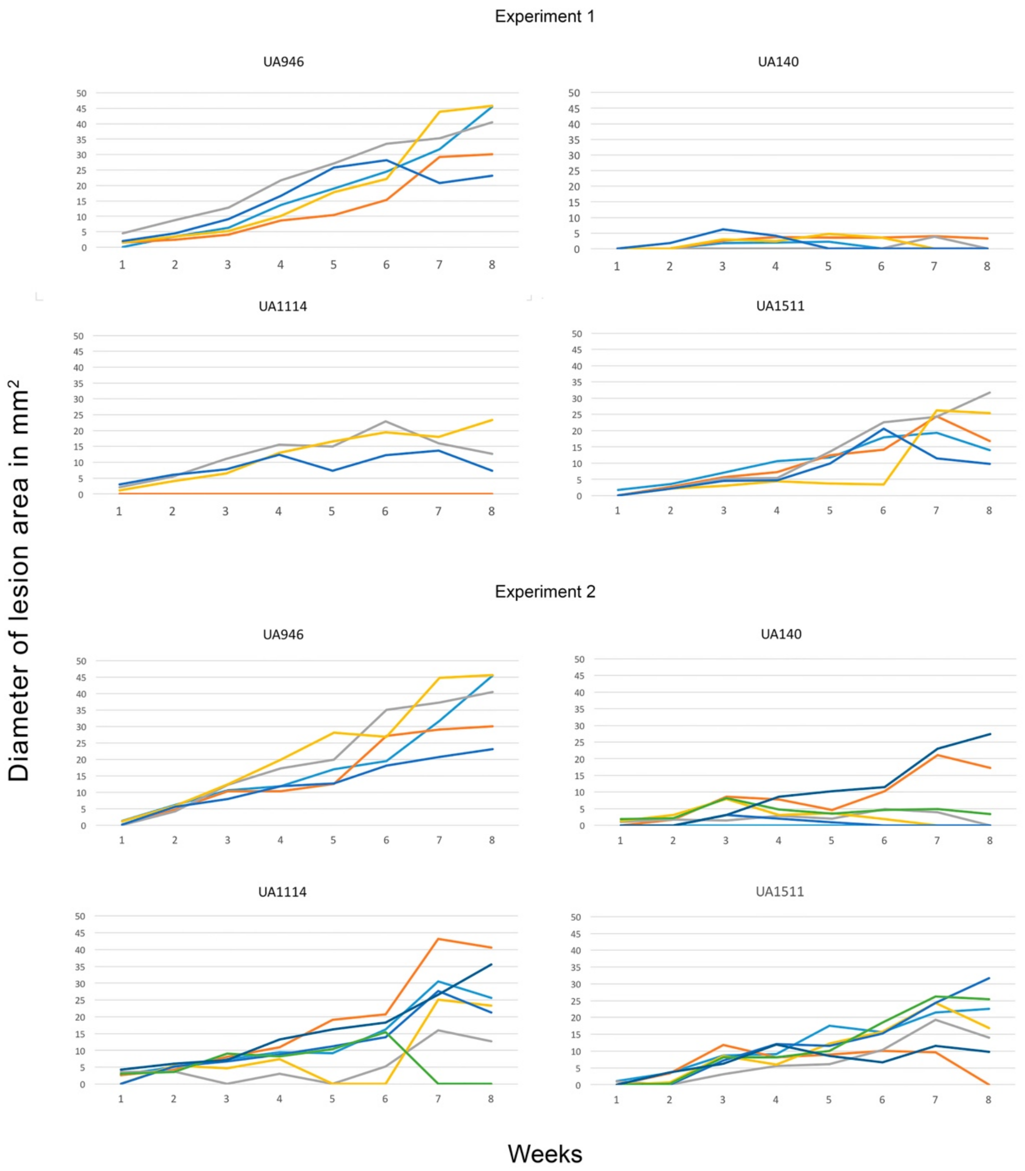

Supplementary Figure 1. Row data of lesion measure in the ear per week for each strain in both experiment done. Each line represents the follow performed on each BALB/c mice.

Lesion area measure

Kruskal-Wallis test for equal medians

H (chi2): 69,31
Hc (tie corrected): 69,99
p (same): 4,28E-15
There is a significant difference between sample medians

Mann-Whitney pairwise comparisons with Bonferroni correction of p values

	UA946	UA140	UA1114	UA1511
**UA946**		9,88E-14	0,004352	0,002041
**UA140**	9,88E-14		2,87E-06	3,59E-08
**UA1114**	0,004352	2,87E-06		1
**UA1511**	0,002041	3,59E-08	1	

Lesion intensity scale

Kruskal-Wallis test for equal medians

H (chi2): 42,41
Hc (tie corrected): 46,32
p (same): 4,85E-10
There is a significant difference between sample medians

Mann-Whitney pairwise comparisons with Bonferroni correction of p values

	UA946	UA140	UA1114	UA1511
**UA946**		1,52E-08	0,002517	0,006404
**UA140**	1,52E-08		0,0001386	0,0001283
**UA1114**	0,002517	0,0001386		1
**UA1511**	0,006404	0,0001283	1	

Parasitic load

Kruskal-Wallis test for equal medians

H (chi2): 8,314
Hc (tie corrected): 8,862
p (same): 0,03118
There is a significant difference between sample medians

Mann-Whitney pairwise comparisons with Bonferroni correction of p values

	UA946	UA140	UA1114	UA1511
**UA946**		0,02173	0,09283	0,08055
**UA140**	0,02173		0,07839	0,1051
**UA1114**	0,09283	0,07839		0,6916
**UA1511**	0,08055	0,1051	0,6916	

Supplementary Table1. Kruskal-Wallis statistical test and Mann-Whitney pairwise comparisons between experimental variables for the two experiments performed.

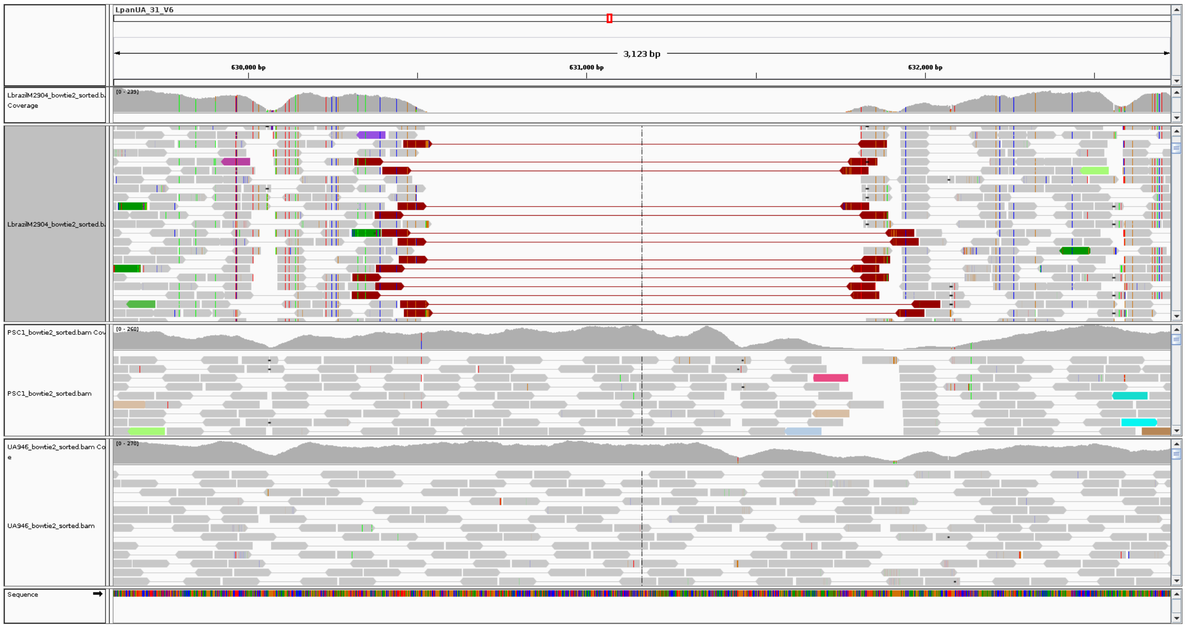

Supplementary Figure 2. Example of visualization of short read alignments for a 1kbp region in which an insertion was predicted within UA946 (lower panel) relative to M2904 (upper panel), using whole genome comparisons. Aligned reads from the *L. panamensis* strain PSC-1 is also shown in the middle panel. Red alignments have predicted insert lengths of more than 1kbp. Reads painted with other colors have their mates aligned to other chromosomes.

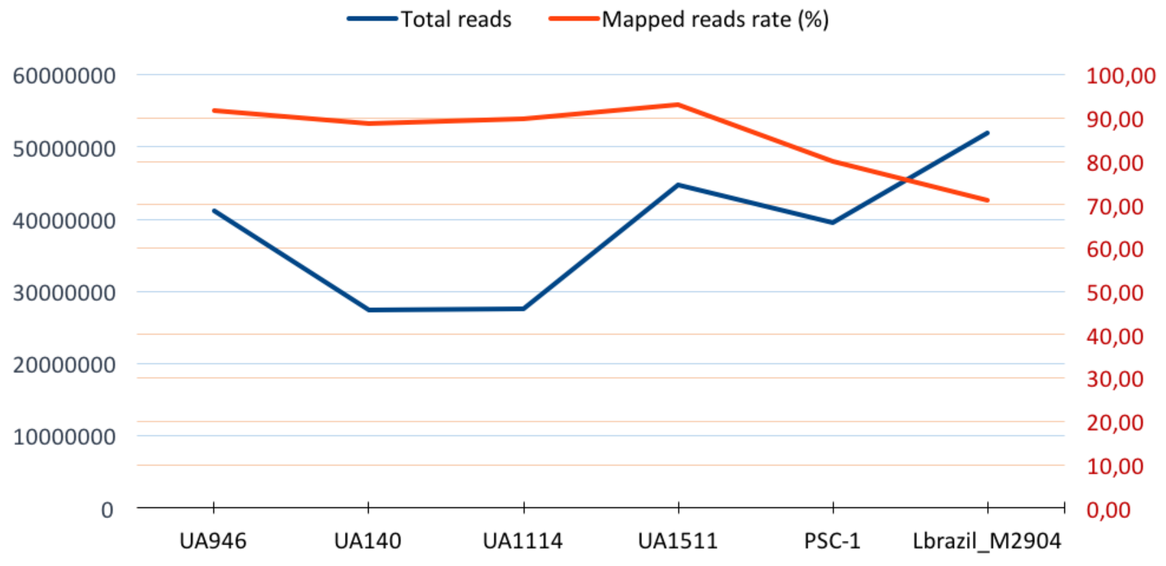

Supplementary Figure 3. Illumina reads mapped to the genome of strain UA946 of *L. panamensis*.

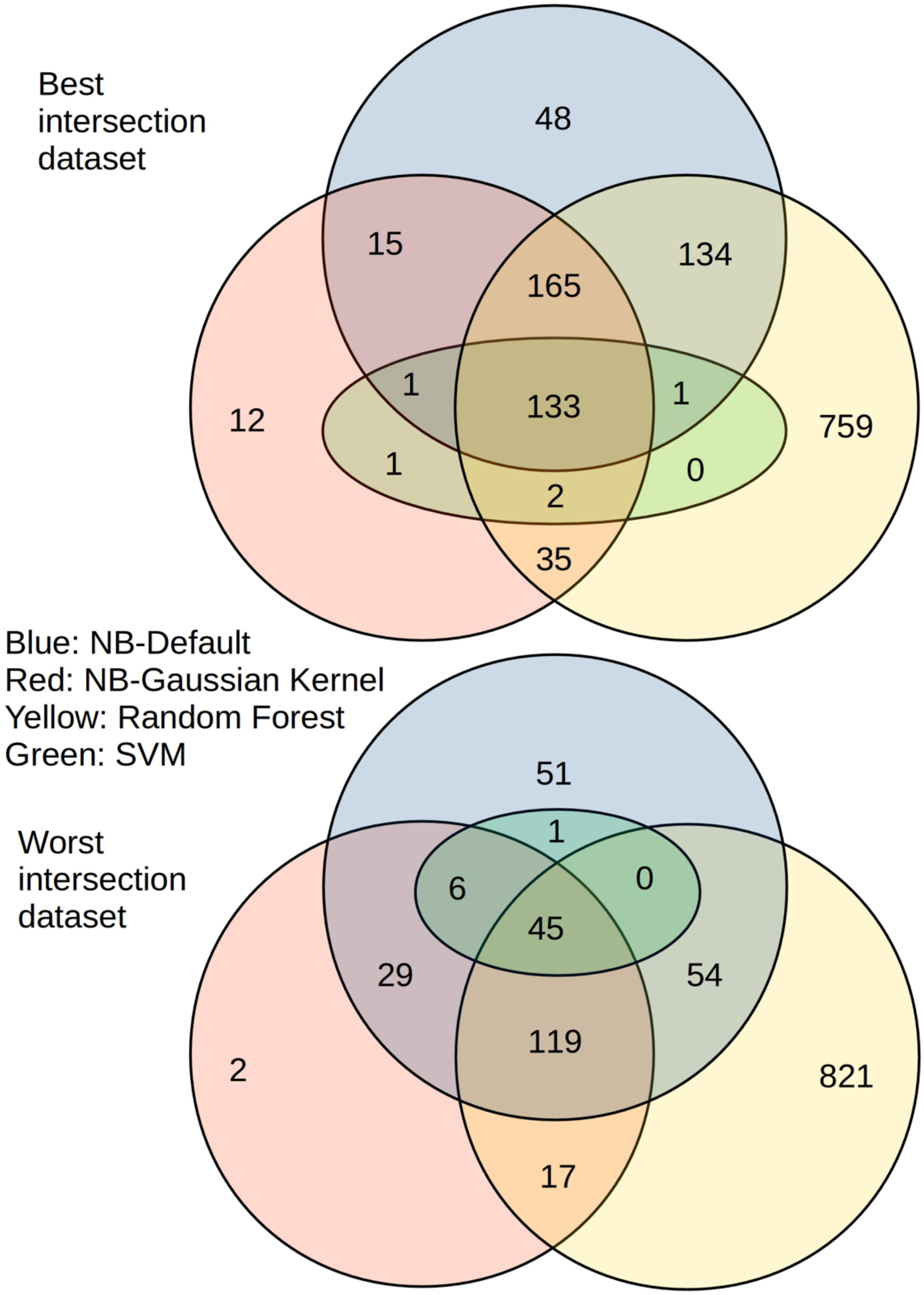

Supplementary Figure 4. Venn diagrams for the scenario in which there is more intersection between machine learning methods and for which there is less intersection.

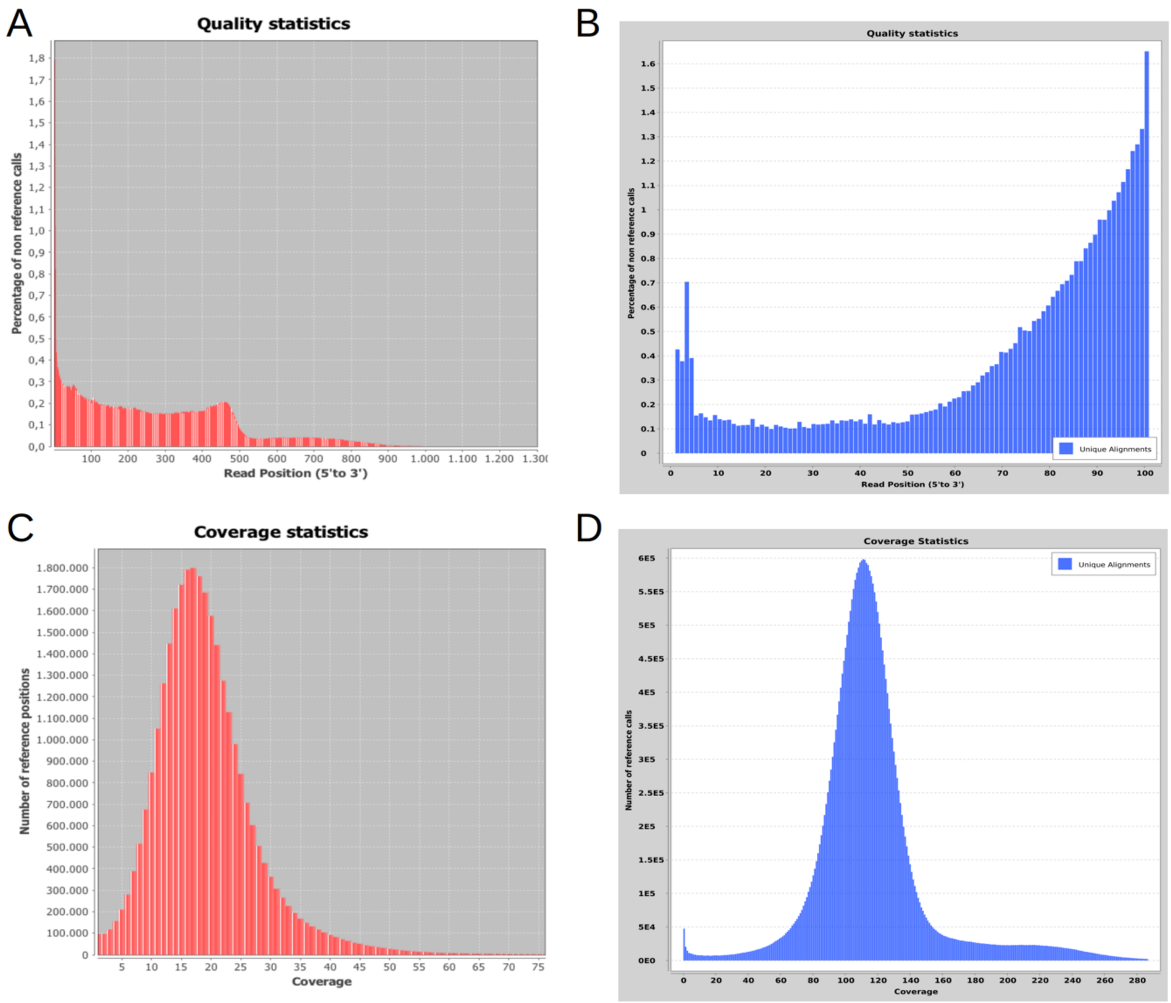

Supplementary Figure 5. Quality test of assembled genome based on the mapping error percentage of reads by position; A) 454 reads. B) Paired-end reads illumina. Media coverage sequencing; C) 454 reads. D) Paired-end reads illumina (program NGSEP).

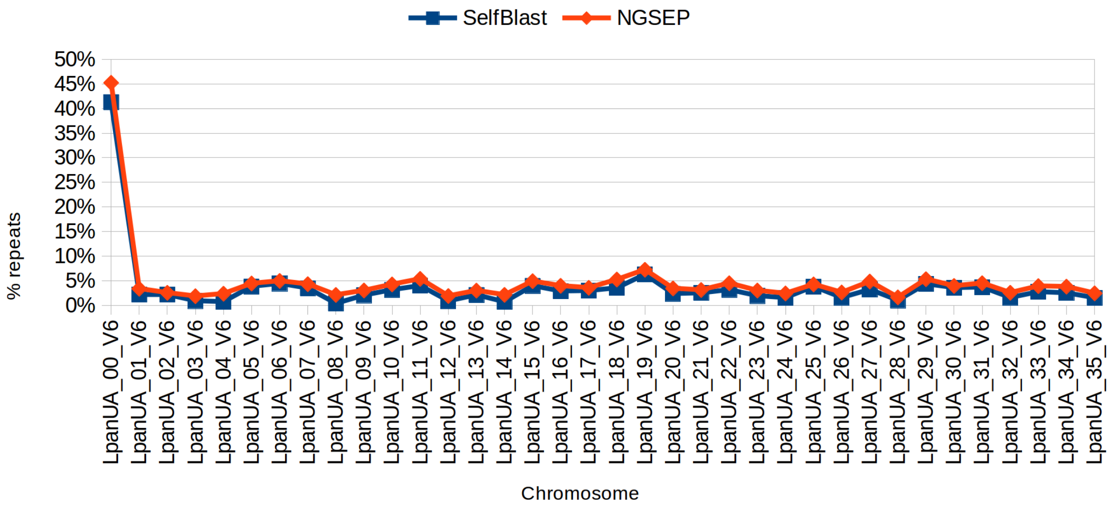

Supplementary Figure 6. Repetitive regions identified by self blast and self mapping using NGSEP program.

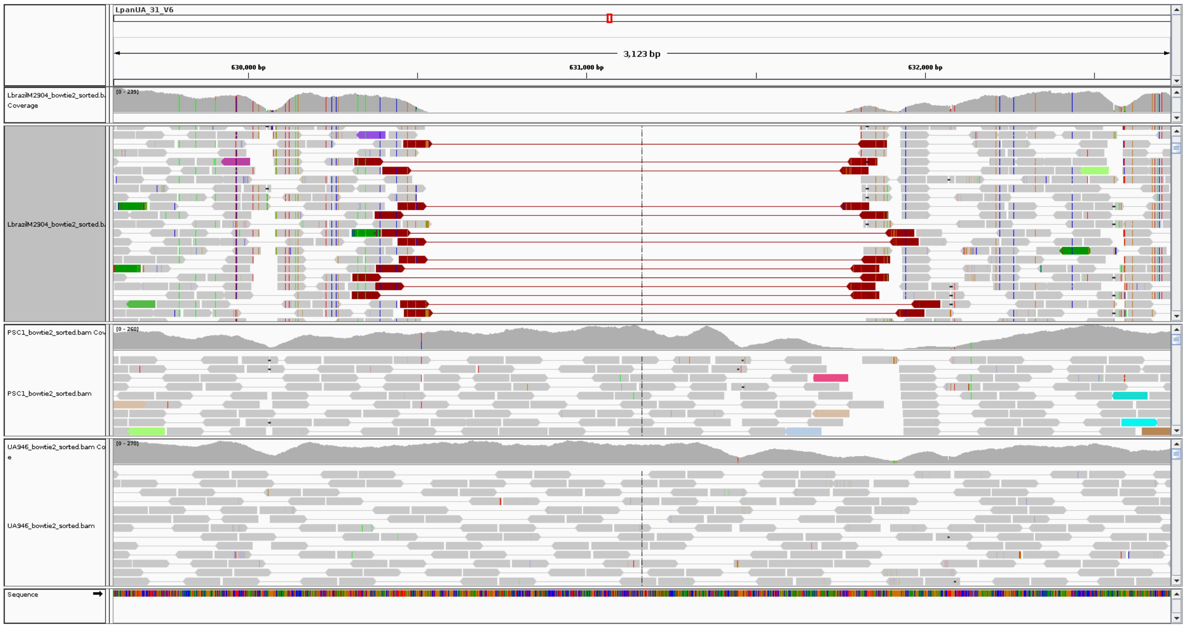

Supplementary Figure 7. Example of visualization of short read alignments for a 1kbp region in which an insertion was predicted within UA946 (lower panel) relative to M2904 (upper panel) using whole genome comparisons. Aligned reads from the *L. panamensis* strain PSC-1 is also shown in the middle panel. Red alignments have predicted insert lengths of more than 1kbp. Reads painted with other colors have their mates aligned to other chromosomes.

Chr	Chromosomes before IMAGE			Chromosomes after IMAGE			Difference		
	Length	Ns	#gaps	Length	Ns	#gaps	Length	Ns	#gaps
1	267.023	10.844	22	267.401	10.775	21	378	69	1
2	296.744	35.551	18	297.007	33.487	18	263	2064	0
3	387.899	10.262	14	389.201	10.216	16	1.302	46	2
4	436.471	9.163	47	436.986	9.040	20	515	123	27
5	476.818	30.324	27	569.282	29.424	22	92.464	900	5
6	516.202	10.246	34	516.730	10.239	18	528	7	16
7	565.104	21.593	19	565.747	19.040	17	643	2553	2
8	446.896	21.292	18	447.970	20.699	15	1074	593	3
9	582.716	32.282	24	584.259	31.633	24	1543	649	0
10	526.398	45.179	16	527.106	43.313	13	708	1866	3
11	592.229	25.477	24	589.864	18.352	20	2365	7125	4
12	493.387	5.804	14	493.739	5.610	14	352	194	0
13	615.759	6.756	16	616.788	6.895	13	1029	139	3
14	651.698	86.728	42	651.698	85.161	17	0	1567	25
15	614.203	19.840	18	614.686	19.422	18	483	418	0
16	692.608	43.832	41	693.464	43.315	26	856	517	15
17	682.174	72.101	18	682.291	71.514	16	117	587	2
18	687.899	17.355	20	690.206	17.239	18	2307	116	2
19	665.187	32.370	19	677.420	32.121	17	12233	249	2
20	2.520.950	230.606	83	2.521.761	222.316	58	811	8290	25
21	748.705	21.347	25	748.814	21.123	16	109	224	9
22	668.618	34.667	55	762.856	32.295	25	94238	2372	30
23	775.951	47.379	44	776.507	42.467	33	556	4912	11
24	823.052	17.427	24	823.589	16.279	22	537	1148	2
25	886.525	11.916	30	888.185	11.400	17	1660	516	13
26	1.026.257	13.038	23	1.026.457	10.427	15	200	2611	8
27	1.032.823	38.679	26	1.033.693	38.123	25	870	556	1
28	1.168.439	34.150	27	1.170.028	34.428	23	1589	278	5
29	1.212.475	52.225	40	1.214.198	49.541	34	1723	2684	6
30	1.304.861	8.162	21	1.397.265	8.061	18	92404	101	3
31	1.345.191	30.495	19	1.345.688	28.649	17	497	1846	2
32	1.534.377	26.065	40	1.534.710	24.234	21	333	1831	19
33	1.440.683	55.622	46	1.441.563	54.233	32	880	1389	14
34	1.986.720	71.843	60	1.985.663	65.598	49	1057	6245	11
35	2.619.390	67.568	116	2.621.291	55.289	93	1901	12279	23
Total	31.292.432	1.298.188	1130	31.604.113	1.231.958	841	318.525	67.064	294

Supplementary Table2. Use of the IMAGE tool for the closure of gaps in the genome of strain UA946 of *L. panamensis*.

Geographical origin	Old World			New World				
Clinical form	VL		CL	CL / DCL		CL / MCL		
Subgenus	*Leishmania*					*Viannia*		
Species	*L. infantum*	*L. donovani*	*L. major*	*L. mexicana*	*L. amazonensis*	*L. braziliensis*	*L. guyanensis*	*L. naiffi*
Strain	JPCM5	BPK282/0cl4-LV9	Friedlin	U1103	M2269	M2904	LgCL085	LnCL223
Sequencing	Sanger/Illumina	454/Illumina	Sanger/Illumina	Sanger/Illumina	454/Illumina	Sanger/Illumina	Illumina	Illumina
Size	32,101,728	32,444,998	32,855,089	32,108,741	32,444,998	31,997,773	31,014,322	30,341,095
Contigs/scaffolds	562	2.154	36	375	2.627	1.041	2.800	6.530
Chromosomes	36	36	36	34	34	35	35	35
Depth coverage	80X	74X	56X	112X	74X	107X	56X	36X
Heterozygous SNPs	629	3549	297	12531	N/A	44588	705	14739
Overall GC content (%)	59,6	57,3	59,7	59,7	58,5	57,8	57,34	57,53
Coding GC content (%)	62.4	61	62.5	61.23	61	60.3	N/A	N/A
Genes	8241	8252	8412	8250	8100	8359	8376	8262
Pseudogenes	41	N/A	97	98	36	195	51	59
Reference	Peacock et al 2007<br>Rogers et al 2011	Downing et al 2011<br>Rogers et al 2011	Peacock et al 2007<br>Rogers et al 2011	Tschoeke et al2014<br>Rogers et al 2011	Tschoeke et al2014<br>Real et al 2015	Peacock et al 2007<br>Rogers et al 2011	Coughlan et al 2018	Coughlan et al 2018

VL (visceral leishmaniases), CL (cutaneous leishmaniases), DCL (diffuse cutaneous leishmaniasis), MCL (mucocutaneous leishmaniasis)

N/A, not available

Supplementary Table3. Genomic characteristics of the strains of *Leishmania* spp., pathogenic for humans reported. The gray shadow shows the contribution generated in this work with the virulent strain UA946 of *L. panamensis*.
